# Stabilising sleep for patients admitted at acute crisis to a psychiatric hospital (OWLS): an assessor-blind pilot randomised controlled trial

**DOI:** 10.1017/S0033291717003191

**Published:** 2017-11-07

**Authors:** Bryony Sheaves, Daniel Freeman, Louise Isham, Josephine McInerney, Alecia Nickless, Ly-Mee Yu, Stephanie Rek, Jonathan Bradley, Sarah Reeve, Caroline Attard, Colin A. Espie, Russell Foster, Anna Wirz-Justice, Eleanor Chadwick, Alvaro Barrera

**Affiliations:** 1 Sleep & Circadian Neuroscience Institute (SCNi), Department of Psychiatry, Warneford Hospital, University of Oxford, Oxford, OX3 7JX, UK; 2 Oxford Health NHS Foundation Trust, Warneford Hospital, Oxford, OX3 7JX, UK; 3 Department of Psychiatry, Warneford Hospital, University of Oxford, Oxford, OX3 7JX, UK; 4 Primary Care Clinical Trials Unit, Nuffield Department of Primary Care Health Sciences, University of Oxford, Radcliffe Primary Care Building, Radcliffe Observatory Quarter, Woodstock Road, Oxford, OX2 6GG, UK; 5 Berkshire Healthcare NHS Foundation Trust, Prospect Park Hospital, Honey End Lane, Tilehurst, Reading, Berkshire, RG30 4EJ, UK; 6 Sleep & Circadian Neuroscience Institute (SCNi), Nuffield Department of Clinical Neurosciences, OMPI, Sir William Dunn School of Pathology, University of Oxford, South Parks Road, Oxford, OX1 3RE, UK; 7 Centre for Chronobiology, Psychiatric Hospital, University of Basel, Wilhelm Klein Strasse 27, CH-4012 Basel, Switzerland; 8 Oxford Health NHS Foundation Trust, Warneford Hospital, Oxford, OX3 7JX, UK

**Keywords:** Bipolar disorder, inpatient, insomnia, psychiatric ward, psychosis, schizophrenia, sleep

## Abstract

**Background:**

When patients are admitted onto psychiatric wards, sleep problems are highly prevalent. We carried out the first trial testing a psychological sleep treatment at acute admission (Oxford Ward sLeep Solution, OWLS).

**Methods:**

This assessor-blind parallel-group pilot trial randomised patients to receive sleep treatment at acute crisis [STAC, plus standard care (SC)], or SC alone (1 : 1). STAC included cognitive–behavioural therapy (CBT) for insomnia, sleep monitoring and light/dark exposure for circadian entrainment, delivered over 2 weeks. Assessments took place at 0, 2, 4 and 12 weeks. Feasibility outcomes assessed recruitment, retention of participants and uptake of the therapy. Primary efficacy outcomes were the Insomnia Severity Index and Warwick–Edinburgh Mental Wellbeing Scale at week 2. Analyses were intention-to-treat, estimating treatment effect with 95% confidence intervals.

**Results:**

Between October 2015 and July 2016, 40 participants were recruited (from 43 assessed eligible). All participants offered STAC completed treatment (mean sessions received = 8.6, s.d. = 1.5). All participants completed the primary end point. Compared with SC, STAC led to large effect size (ES) reductions in insomnia at week 2 (adjusted mean difference −4.6, 95% CI −7.7 to −1.4, ES −0.9), a small improvement in psychological wellbeing (adjusted mean difference 3.7, 95% CI −2.8 to 10.1, ES 0.3) and patients were discharged 8.5 days earlier. One patient in the STAC group had an adverse event, unrelated to participation.

**Conclusions:**

In this challenging environment for research, the trial was feasible. Therapy uptake was high. STAC may be a highly effective treatment for sleep disturbance on wards with potential wider benefits on wellbeing and admission length.

## Introduction

It is almost ubiquitous for patients admitted at acute crisis to a psychiatric hospital to have sleep disturbance. Around eight out of 10 patients report clinically significant insomnia (Haynes *et al.* 2011). A negative correlation has been found between sleep duration at admission to a psychiatric ward and subsequent length of time in hospital (Langsrud *et al.* 2016). There is increasing awareness of the importance of sleep to mental health (Boyce, 2015; Freeman *et al.* 2017). Whilst previously subsumed as a symptom of mental illness, changes in classification now recommend diagnosing and treating sleep disorders (e.g. insomnia) as an independent clinical problem (American Psychiatric Association, 2013). Treatment of insomnia has been shown to lessen psychotic experiences (Freeman *et al.* 2017), mania (Harvey *et al.* 2015), depression (Manber *et al.* 2008; Ye *et al.* 2015) and anxiety (Espie *et al.* 2012; Ye *et al.* 2015). Treatment of insomnia in psychiatric inpatients may therefore be an important clinical target to aid recovery, irrespective of psychiatric diagnosis.

Psychiatric wards bring unique challenges to the sleep system. Staff risk management observations of patients often require a light to be turned on periodically throughout the sleep period. The environment can be noisy. In the daytime, limited access to natural daylight leaves the circadian system vulnerable to dysregulation. Stress from being held in hospital under a section of the Mental Health Act or a coercive route to hospital may exacerbate night-time hyper-arousal. Stabilising sleep may bring benefits for recovery, but interventions such as cognitive–behavioural therapy (CBT) for insomnia require adapting to manage these challenges.

CBT is associated with moderate-to-large effect size improvements in insomnia symptoms (Irwin *et al.* 2006) and is the recommended first-line treatment for persistent insomnia in international clinical guidelines (National Institute for Health and Care Excellence, 2015; Qaseem *et al.* 2016). Two pilot randomised controlled trials (RCTs) have shown positive results for adapted CBT for insomnia protocols in populations with severe mental illness. Large effect size improvements in insomnia symptoms were found in patients with persistent delusions and hallucinations in the context of schizophrenia and related diagnoses (Freeman *et al.* 2015). This protocol particularly took into account circadian rhythm disruption, which is common in patients with schizophrenia (Waite *et al.* 2015). Similarly, offering adapted CBT for insomnia to patients in the euthymic phase of bipolar affective disorder led to reduced insomnia symptoms and fewer days in a bipolar episode (Harvey *et al.* 2015). However, CBT for insomnia has not yet been adapted or tested for patients experiencing an acute episode of severe mental illness whilst in hospital.

Increasing the effectiveness of inpatient treatment works towards the goal of each admission being as ‘short as possible, minimising disruption to life, as well as cost’ (Crisp, 2015). In the UK, inpatient services are the highest area of spending, using over £1 billion of the £5.5 billion budget for adult mental health (Mental Health Strategies, 2012). Despite this spending, there is continued pressure for acute inpatient care beds. The average ward in England is running over maximum capacity (Crisp, 2015). This results in patients travelling long distances from home to receive treatment (Crisp *et al.* 2016). Targeted interventions that reduce the length of an admission would have clear benefits for both patients and services.

The current study builds upon the work testing adapted protocols for treating insomnia in patients experiencing psychosis (Freeman *et al.* 2015) and bipolar disorder (Harvey *et al.* 2015) and uncontrolled studies in inpatient settings (Morin *et al.* 1990; Haynes *et al.* 2011; Breitstein *et al.* 2014). Our sleep treatment at acute crisis (STAC) included CBT for insomnia with three adaptations: (i) enhanced light/dark exposure to stabilise circadian rhythms, (ii) discussion of sleep and activity levels, monitored using ambulatory devices, to engage patients in their treatment and boost motivation and (iii) delivery of the intervention within a 2-week window to ensure all patients receive help.

The study was designed as a pilot RCT. The primary objective was to assess trial procedures on an inpatient ward. Specifically, the aim was to assess recruitment and retention rates, and uptake of the therapy. The secondary objective was to estimate the treatment effect and confidence intervals, compared with standard care (SC) to inform future trials. The sleep treatment was expected to result in quicker and fuller recovery from insomnia and enhance psychological wellbeing.

## Methods

### Study design and participants

This parallel-group assessor-blind pilot RCT tested STAC, in addition to SC, *v.* SC alone. Recruitment took place on one 18-bed male only psychiatric inpatient ward (Vaughan Thomas ward) in the Oxford Health National Health Service (NHS) Foundation Trust, UK. The ward treats adult men, the majority of whom are admitted during an acute episode of psychosis or bipolar affective disorder. Patients sleep in individual bedrooms and are monitored throughout the night by the staff to ensure their safety. The frequency varies from hourly to constant observation, dependent on the individual assessment of risk. Study inclusion criteria were: self-reported symptoms of insomnia [a score of 8 on the Insomnia Severity Index (ISI)], wanting help to improve sleep, willing and able to give informed consent to participate (assessed according to the Mental Capacity Act, 2005) and willing to allow the community care team to be notified of trial participation. Exclusion criteria were: a planned discharge or transfer date within 14 days of the baseline, the patient's home residence was outside the geographical area covered by the Oxford Health NHS Foundation Trust, a command of English language inadequate for psychological therapy or completing assessments and a diagnosis of learning disability or organic syndrome. Patient enrolment was conducted by one graduate psychologist (JM). The week 2 assessment was the primary end point. No changes were made to the design or outcome measures after commencement of the trial.

The trial received ethical approval from the NHS Research Ethics Committee East Midlands, Leicester – Central (15/EM/0341). The trial was registered (ISRCTN15324584). All patients provided written informed consent.

### Randomisation and blinding

Patients were randomly assigned to receive either STAC in addition to SC or SC alone (1 : 1). The randomisation process was made clear to patients in the participant information sheet, prior to the consent process. The randomisation schedule was developed by the University of Oxford Primary Care Clinical Trials Unit using a web-based randomisation system. Minimisation randomisation was used with an 80% chance of selecting the minimising group. Minimisation balanced groups by stratifying on the ISI (symptoms of insomnia, score 0–14 *v.* insomnia disorder, score >14), the Warwick–Edinburgh Mental Wellbeing Scale (WEMWBS; low wellbeing, score 0–35 *v.* high wellbeing, score 36–70) and participant diagnosis made by the ward consultant psychiatrist (non-affective psychotic disorder, affective disorder or ‘other’).

Research assessors (JM and StR) were blind to treatment allocation. The trial therapists (LI, BS and JB) informed the patients of the outcome of randomisation to maintain allocation concealment. Precautionary measures to prevent unblinding included research assessors and trial therapists block booking separate times to be on the ward, patients were reminded about the importance of unbiased assessments (blinding), research assessors did not look at the patient clinical notes after randomisation had taken place and the ward staff were regularly reminded of the importance of blinding via meetings, posters and leaflets. In the case of an unblinding, an alternative research assessor (blind to allocation) was used. This happened nine times in total, eight times at the 2-week assessment and once at the 4-week assessment. Every assessment was completed by an assessor who was blind to treatment allocation. Measures were also taken to prevent contamination between the intervention and control groups. Participants in the intervention group were asked not to discuss the therapy with other patients and the staff were also told about the potential implications of contamination for the trial results.

### Procedures

STAC was provided by a clinical psychologist (LI, BS or JB) in one-to-one sessions with each patient. Treatment took place on the ward, in a local clinic room, or the local community whilst using ward leave. BS and DF provided supervision. STAC was delivered over a 2-week therapy window. Five sessions were defined as a minimum dose (to include both formulation and completion of at least one active therapy technique). The frequency and duration of sessions were flexible depending on the patient preference and clinical presentation.

The key therapeutic techniques were adapted CBT for insomnia. This was supplemented with light/dark exposure for circadian entrainment. Wrist-worn fitness trackers (Basis Peak watch) were used as a therapy tool for assessment, to inform the development of a collaborative sleep plan and to boost motivation. These watches report individual sleep periods, activity levels and heart rate, which are synced to an iOS application. The CBT for insomnia treatment techniques was taken from three main sources (Espie, 2010; Kaplan & Harvey, 2013; Waite *et al.* 2015). The intervention was written into manual style booklets. These were shared with the patients. One manual was used to guide each treatment session. Session 1 included psychoeducation, assessment and goal setting. The focus of subsequent sessions was chosen based on the key maintenance factors identified in session 1. Treatment techniques included: setting a consistent sleep window, stimulus control, boosting circadian zeitgebers (light/dark, meals and timing of activity), wind-down (including relaxation) and rise routines, strategies to manage night-time worry and voices, and sleep hygiene. The final session was always relapse management, including planning sleep strategies for use upon discharge.

Key adaptations to the CBT for insomnia protocol for this population included: (i) a rationale of boosting sleep as a tool for recovery; (ii) delivering the intervention intensively over a 14-day therapy window to ensure that patients received a full dose prior to discharge; (iii) stimulus control was completed within the patient bedrooms, whereby a patient was encouraged to leave the bed if unable to sleep and sit instead on a bean bag; (iv) behavioural experiments to test beliefs about the use of daytime sleep to combat fatigue (common due to sedative medication or depressive symptoms) and (v) practical strategies to reduce the impact of night-time observations from the staff.

Principles of sleep restriction informed the intervention. Time spent in bed was limited (particularly during the daytime and early evening), and a consistent sleep window was collaboratively set to ensure optimal sleep pressure each night. However, this was achieved by setting the goal sleep window (often 7–8 h) and ensuring that the window was timed in line with the circadian preference (i.e. a ‘morning type’ typically had an earlier sleep window than an ‘evening type’). The sleep window was *not* set to less than the goal sleep duration.

Exposure to light/dark therapy was used according to a clinical manual (Wirz-Justice *et al.* 2013). We used light to shift circadian rhythms and/or boost morning arousal. Our preference was for timed exposure to natural daylight outdoors. Where access to natural daylight was not possible, due to patient illness or restricted ward leave, we used Lumie Brazil light boxes, which emit 10 000 lux of light at a distance of 35 cm. Low light levels in the evening period were emphasised for all patients to strengthen circadian rhythms, but particularly for patients with a diagnosis of bipolar affective disorder, given the known sensitivity to light in this group (Barbini *et al.* 2005). A detailed description of the therapy is available elsewhere (Sheaves *et al.* in press).

SC was delivered according to the national and local protocols and guidelines. This typically included medication and contact with full-time psychiatry, nursing, occupational therapy, social work and health care assistant staff. A clinical psychologist offered staff support and patient sessions 1 day per week. Patients were invited to weekly multi-disciplinary ward round meetings.

### Outcomes

The primary objective of the Oxford Ward sLeep Solution (OWLS) was to assess the feasibility of trial procedures. Feasibility outcomes included the percentage of patients admitted to the ward who were recruited during the trial period, retention of participants and uptake of the therapy. The primary efficacy outcome measures were the ISI (Bastien *et al.* 2001) and the WEMWBS(Tennant *et al.* 2007). Higher scores on the ISI indicate more severe insomnia. The internal consistency (Cronbach's *α* at baseline) of the ISI in the present study was 0.78. Higher scores on the WEMWBS indicate higher psychological wellbeing. The internal consistency of the WEMWBS in the present study was 0.95.

Secondary efficacy outcome measures included interviewer-rated assessments of psychiatric symptoms [Positive and Negative Syndromes Scale (PANSS, Kay *et al.* 1987) and Young Mania Rating Scale (YMRS, Young *et al.* 1978)]. The internal consistency of these scales in the present study were PANSS positive 0.59, PANSS negative 0.60, PANSS general 0.62 and YMRS 0.66. Global distress was assessed via a ten 10- self-report measure [Clinical Outcomes in Routine Evaluation, (CORE-10, Connell & Barkham, 2007)]. The internal consistency of the CORE-10 in the present study was 0.86. Suicidal ideation was assessed via the Beck Suicide Scale (BSS, Beck *et al.*, 1979). The internal consistency of the BSS in the present study was 0.81.

Tertiary efficacy outcome measures included health-related quality of life (EQ-5D) (The EuroQol Group, 1990). The internal consistency of the EQ-5D in the present study was 0.56. Patient satisfaction was assessed by an adapted version of the Client Satisfaction Questionnaire (Larsen *et al.* 1979) at the end of the therapy. Each item was rated on a five- point Likert scale. Higher scores indicate higher satisfaction with therapy.

Provision of SC was monitored via medical records for each patient using a modified version of the Client Service Receipt Inventory (Beecham & Knapp, 1992). This included the duration of admission between baseline and the 12-week assessment date (taken from patients’ medical records) and medication at each assessment. The defined daily dose (DDD) (World Health Organization Collaborating Centre for Drug Statistics Methodology, 2016) was used to convert all anti-psychotic, mood stabiliser and anxiolytic medications into an equivalent dose for each patient. The DDD is the gold standard measure for comparing drug utilisation (World Health Organization Collaborating Centre for Drug Statistics Methodology, 2016). The number of pro re nata (PRN, ‘taken when necessary’) medications prescribed were also measured across groups.

Serious adverse events (SAEs) were defined as (i) deaths, (ii) suicide attempts, (iii) serious violent incidents, (iv) admissions to secure units and (v) formal complaints about the therapy. If the research team became aware of an adverse event, it was reviewed and reported to the Oxford Health NHS Foundation Trust Trial Safety Review Group (TSRG). This group is independent from the research team. The TSRG determined whether or not the SAE was related to participation in the trial. Upon completion of the trial, the medical records of all participants were reviewed for SAEs, which had not been brought to the attention of the trial team.

### Statistical analysis

A detailed statistical analysis plan (SAP) was completed prior to conducting the analysis. A sample size of 30 has been recommended for use in pilot studies where the treatment effect is expected to be large (Browne, 1995). Accounting for dropout, we estimated that 50 participants would be required to meet the trial aims, as has been used in a previous study of insomnia treatment for those with psychosis (Freeman *et al.* 2015).

Visual analysis of the histograms for the residuals of all efficacy outcome measures was assessed and deemed sufficiently normal for each subsequent analysis. Adjusted treatment difference and confidence intervals were estimated using a linear mixed-effects model, which accounts for repeated measures over time. Baseline score of the outcome and the stratification variables (diagnosis and baseline scores for insomnia and wellbeing) were added as covariates in the model. Assessment point (weeks 2, 4 and 12), outcome of randomisation and an interaction between assessment point and randomised group were included as fixed effects to allow estimation of the treatment effect at the three time points. Repeated measurements were accounted for by fitting random intercepts for each participant. Given the objectives of this pilot RCT, the analysis plan did not include reporting of *p* values. A sensitivity analysis was planned for the primary efficacy outcome measures, adding baseline duration of hospital admission as a covariate. Sub-group analyses were pre-specified in the SAP, to assess the impact of acute manic symptoms at baseline on self-reported outcomes (online Supplementary material). Owing to small sample sizes, these report descriptive statistics only.

The duration of time in hospital was analysed using analysis of covariance, given there was no repeated measurement. Baseline duration in hospital and stratification factors (diagnosis and baseline scores for insomnia and wellbeing) were added as covariates.

Standardised effect sizes are reported using Cohen's *d* (adjusted mean difference between groups/pooled baseline standard deviation). Analysis was conducted after the last trial assessment was complete and followed intention-to-treat principles. Analyses were conducted by BS using SPSS for windows version 23 (IBM Corp, 2015) and validated by a trial statistician (AN).

## Results

**Fig. 1.**
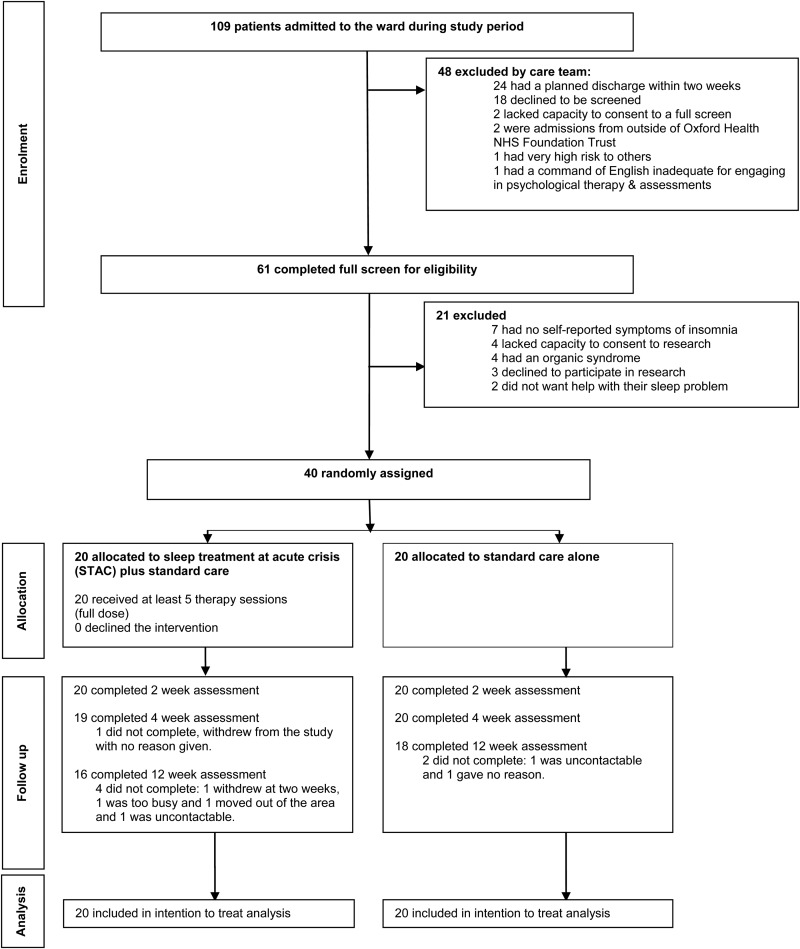
Flow diagram of trial participants.

Recruitment took place over 9 months (October 2015 to July 2016), with a 4-week break to account for therapist leave. During the recruitment period, 40 patients were randomly allocated to receive STAC in addition to SC (*n* = 20) or SC alone (*n* = 20). The target of 50 patients was not reached owing to lower throughput of patients on the ward. Figure 1 shows the flow of participants through the trial. All 40 participants completed the baseline and 2-week assessments (primary end point). Thirty-four participants completed all assessments (weeks 0, 2, 4 and 12) for the primary efficacy measures (85%). One participant dropped out at week 4 and a further five dropped out at the week 12 assessment (Fig. 1). In the STAC group, all participants (*n* = 20, 100%) completed the intervention. The mean number of treatment sessions received across the 14-day therapy window was 8.6 (s.d. = 1.5) and the mean duration of sessions was 44.8 min (s.d. = 15.6). No more than one therapy session was offered in a day and the aim was to provide more sessions in week 1 of the therapy and taper sessions in week 2. Across the STAC group, 171 sessions were attended out of 187 offered (91.4%). Of the 16 sessions not attended, 10 (62.5%) were due to service-related factors (e.g. clashes with tribunals, Mental Health Act assessments or with escorted leave availability) and six (37.5%) were due to patient factors (e.g. the patient feeling too unwell to attend).

Baseline and clinical characteristics were largely similar across the two randomised groups (Table 1). The average age was 40 years, similar to other studies recruiting those with severe mental illnesses (Freeman *et al.* 2015; Harvey *et al.* 2015). Most participants were single, unemployed prior to admission, detained under a section of the Mental Health Act (1983) and a quarter had no current accommodation. With regards to chronotype, there were a higher number of patients in the SC group who fell into the ‘moderate morning’ group, and more patients in the STAC group who fell into the ‘intermediate’ chronotype group.

**Table 1.** Baseline demographics and clinical characteristics (*N* = 40)

	Sleep treatment at acute crisis (*n* = 20)	Standard care alone (*n* = 20)
Age (years)	40 (12)	40 (14)
Ethnicity		
White British	12 (60%)	15 (75%)
White other	3 (15%)	3 (15%)
Mixed ethnic group	1 (5%)	1 (5%)
Asian	2 (10%)	1 (5%)
Black (African)	2 (10%)	0 (0%)
Marital status		
Single	14 (70%)	13 (65%)
Married	4 (20%)	2 (10%)
Cohabiting	2 (10%)	1 (5%)
Divorced/separated	0 (0%)	4 (20%)
Employment status prior to admission		
Unemployed	11 (55%)	9 (45%)
Registered sick	4 (20%)	4 (20%)
Employed full time	2 (10%)	4 (20%)
Student	2 (10%)	0 (0%)
Volunteer	0 (0%)	2 (10%)
Retired	1 (5%)	0 (0%)
Full-time carer	0 (0%)	1 (5%)
Accommodation status		
No fixed abode (awaiting accommodation)	4 (20%)	6 (30%)
Living alone	5 (25%)	4 (20%)
Living with others	2 (10%)	5 (25%)
Living with parents	4 (20%)	1 (5%)
Living with spouse/partner	5 (25%)	2 (10%)
Living with other relatives	0 (0%)	2 (10%)
Diagnosis		
Schizophrenia spectrum and other psychotic disorders	9 (45%)	9 (45%)
Affective disorders	11 (55%)	11 (55%)
Bipolar affective disorder	7 (35%)	4 (20%)
Depressive episode/disorder	4 (20%)	6 (30%)
Generalised anxiety disorder	0 (0%)	1 (5%)
Mental Health Act (1983) status at baseline		
Informal admission	7 (35%)	4 (20%)
Section 2 (for assessment)	5 (25%)	8 (40%)
Section 3 (for treatment)	8 (40%)	8 (40%)
Insomnia severity (ISI)		
Symptoms of insomnia (8–14)	6 (30%)	7 (35%)
Moderate insomnia (15–21)	9 (45%)	9 (45%)
Severe insomnia (22–28)	5 (25%)	4 (20%)
Wellbeing (WEMWBS)	39.8 (15.4)	42.3 (13.1)
Chronotype (MEQ)		
Moderate morning	3 (15%)	8 (40%)
Intermediate	11 (55%)	5 (25%)
Moderate evening	6 (30%)	7 (35%)
Number of previous admissions over past 5 years	0.6 (0.7)	1.1 (1.7)
Total number of days in hospital over past 5 years (median, IQR)	2.5 (0,21)	5.5 (0,49)
Duration of index admission up until baseline (median, IQR)	6 (4,26)	9 (5,18)

Data are: *n* (%) or mean (s.d.) unless otherwise indicated.

CBT, cognitive–behavioural therapy; ISI, Insomnia Severity Index; WEMWBS, Warwick-Edinburgh Mental Wellbeing Scale; MEQ, Morningness-Eveningness questionnaire.

Provision of SC (Table 2) and prescribed medication (Table 3) were similar at all assessment points. Polypharmacy was the norm; the mean number of medications was similar across both groups at baseline (mean = 2.5, s.d. = 1.3 for the STAC group and mean = 2.3, s.d. = 1.1 for the SC group). This reduced marginally by the 12-week assessment (mean = 2.1, s.d. = 1.1 for the STAC group and mean = 2.0, s.d. = 1.1 in the SC group). At baseline, less than half of the participants were prescribed PRN hypnotic medication (STAC group *n* = 7, SC alone group *n* = 6). At week 2, five patients in each trial group were prescribed PRN medications. This decreased by the 4-week assessment (STAC group *n* = 4, SC alone group *n* = 3) and again at the 12-week assessment (STAC group *n* = 0, SC alone group *n* = 1). All PRN medications are reported in online Supplementary Table S1.

**Table 2.** Use of standard National Health Service (NHS) care

	*n*	Sleep treatment at acute crisis	*n*	Standard care alone group
Contacts with community psychiatrist				
6 months prior to baseline	20	4.4 (2.6)	20	4.4 (3.5)
Week 2	20	0.3 (0.7)	20	0.2 (0.5)
Week 4	20	0.2 (0.4)	20	0.3 (0.5)
Week 12	18	1.3 (2.2)	20	0.9 (1.8)
Contacts with care co-ordinator				
6 months prior to baseline	20	6.9 (4.6)	20	6.9 (8.2)
Week 2	20	2.3 (4.2)	20	0.8 (0.8)
Week 4	20	1.5 (1.4)	20	1.2 (1.4)
Week 12	18	3.8 (4.6)	20	4.1 (2.3)
Contacts with psychologist (outside of the trial)				
6 months prior to baseline	20	0.1 (0.3)	20	0.3 (0.7)
Week 2	20	0.5 (0.7)	20	0.4 (0.6)
Week 4	20	0.3 (0.6)	20	0.3 (0.5)
Week 12	18	0.2 (0.5)	20	0.5 (0.7)
Number of days attended day hospital				
6 months prior to baseline	20	1.2 (1.7)	20	1.0 (3.1)
Week 2	20	0.7 (1.3)	20	0.1 (0.4)
Week 4	20	1.2 (2.2)	20	1.2 (2.2)
Week 12	18	1.3 (1.9)	20	2.7 (4.5)
Subsequent psychiatric inpatient admission within 12 weeks (i.e. relapse following discharge)	18	0 (0%)	20	1 (5%)

Data are mean (s.d.) or *n* (%).

**Table 3.** Medication use over time

	Sleep treatment at acute crisis (*n* = 20)	Standard care alone (*n* = 20)
Number of medications		
Week 0	2.5 (1.3)	2.3 (1.1)
Week 2	2.6 (1.2)	2.3 (1.1)
Week 4	2.5 (1.5)	2.2 (1.2)
Week 12	2.1 (1.1)	2.0 (1.1)
Defined daily dose of anti-psychotics		
Week 0	1.5 (1.9)	0.9 (0.7)
Week 2	1.6 (1.9)	1.5 (2.0)
Week 4	1.0 (0.6)	1.0 (0.6)
Week 12	1.4 (1.3)	1.1 (0.6)
Defined daily dose of mood stabilisers		
Week 0	0.3 (0.1)	0.1 (0.2)
Week 2	0.3 (0.6)	0.1 (0.2)
Week 4	0.2 (0.2)	0.1 (0.2)
Week 12	0.2 (0.5)	0.1 (0.3)
Defined daily dose of anxiolytics		
Week 0	0.1 (0.2)	0.1 (0.2)
Week 2	0.3 (0.7)	0.1 (0.3)
Week 4	0.2 (0.7)	0.1 (0.3)
Week 12	0.0 (0.1)	0.1 (0.2)
Defined daily dose of anti-depressants		
Week 0	0.6 (1.3)	0.9 (1.4)
Week 2	0.9 (1.6)	1.1 (1.6)
Week 4	0.8 (1.5)	1.0 (1.4)
Week 12	0.9 (1.6)	1.0 (1.4)

Defined daily dose = a gold standard equivalence measure of drug utilisation (World Health Organization Collaborating Centre for Drug Statistics Methodology, 2016).

### Primary efficacy outcomes

The STAC group had a treatment benefit in the large effect size range at 2 weeks (Table 4), compared with SC. At 4 weeks, the treatment still conferred a benefit, in the medium effect size range when compared with SC alone.

**Table 4.** Scores for primary efficacy outcome measures

	Sleep treatment at acute crisis (*n* = 20)	Standard care (*n* = 20)	Adjusted mean difference between groups (95% CI)	Between-group standardised effect size (*d*)
*Primary efficacy outcome measures*				
Insomnia (ISI)				
Week 0	17.1 (6.0)	16.1 (4.9)		
Week 2	8.5 (5.4)	12.5 (5.5)	−4.6 (−7.7 to −1.4)	−0.9
Week 4	6.8 (5.2)	10.1 (5.6)	−3.6 (−7.0 to −0.3)	−0.7
Week 12	5.8 (4.9)	8.6 (4.4)	−2.8 (−6.3 to 0.7)	−0.5
Wellbeing (WEMWBS)				
Week 0	39.8 (15.4)	42.3 (13.1)		
Week 2	47.4 (10.5)	44.8 (13.4)	3.7 (−2.8 to 10.1)	0.3
Week 4	48.3 (11.7)	45.6 (10.3)	3.6 (−2.8 to 9.9)	0.3
Week 12	48.3 (12.3)	44.4 (12.9)	4.3 (−4.1 to 12.7)	0.3

Data are mean (s.d.).

ISI, Insomnia Severity Index; WEMWBS, Warwick–Edinburgh Mental Wellbeing Scale.

All analyses controlled for stratification factors (insomnia severity, wellbeing and diagnosis).

At week 0, all patients’ ISI scores indicated clinically significant insomnia symptoms (ISI ⩾ 8). By week 2, eight participants in the STAC group reported no clinically significant insomnia symptoms (40%) compared with four in the SC alone group (20%). A small treatment effect was found on psychological wellbeing at all time points when compared with SC alone. Wide confidence intervals for this effect include the possibility that STAC may increase or decrease psychological wellbeing. Results of the sensitivity analysis on both insomnia and wellbeing (online Supplementary Table S2) showed a similar pattern of results. Planned sub-group analyses (online Supplementary Table S3) revealed that patients presenting with manic symptoms at baseline, and hence elevated baseline wellbeing, may have reduced the treatment effects for wellbeing.

### Secondary efficacy outcomes

For secondary outcomes (Table 5), the effect sizes, in general, were in the direction of STAC improving assessment scores. However, the wide confidence intervals for each variable cover a range from STAC potentially increasing or decreasing scores on psychiatric symptoms (YMRS rated mania, PANSS positive, negative and general psychopathology and suicidal ideation) and duration of admission, when compared with SC alone. Global distress had a small treatment effect at 2 weeks, which was maintained at 4 weeks. By 12 weeks, the SC alone group had scores similar to the treatment group.

**Table 5.** Scores for secondary efficacy outcome measures

	Sleep treatment at acute crisis (*n* = 20)	Standard care (*n* = 20)	Adjusted mean difference between groups (95% CI)	Between-group standardised effect size (*d*)
Positive symptoms (PANSS)				
Week 0	15.3 (6.6)	15.4 (5.2)		
Week 2	12.2 (4.8)	12.5 (4.6)	0.2 (−2.1 to 2.4)	0.0
Week 4	11.2 (3.9)	11.2 (4.3)	0.1 (−2.1 to 2.4)	0.0
Week 12	9.4 (2.9)	10.4 (3.5)	−0.4 (−3.0 to 2.3)	−0.1
Negative symptoms (PANSS)				
Week 0	14.7 (6.0)	13.9 (4.3)		
Week 2	12.8 (4.1)	13.8 (5.7)	−1.4 (−4.3 to 1.4)	−0.3
Week 4	11.9 (4.6)	13.6 (5.1)	−2.0 (−5.3 to 1.2)	−0.4
Week 12	11.9 (3.5)	14.9 (7.4)	−3.7 (−7.8 to 0.3)	−0.7
General psychopathology (PANSS)				
Week 0	38.4 (9.2)	39.2 (8.3)		
Week 2	31.4 (6.6)	34.7 (8.5)	−3.1 (−7.2 to 1.1)	−0.4
Week 4	30.8 (8.8)	30.2 (8.0)	0.8 (−3.8 to 5.5)	0.1
Week 12	29.1 (8.4)	30.5 (11.8)	−0.4 (−7.3 to 6.5)	−0.1
PANSS total				
Week 0	68.3 (16.3)	68.4 (14.9)		
Week 2	56.4 (9.2)	61.1 (15.5)	−4.4 (−11.5 to 2.8)	−0.3
Week 4	53.9 (12.6)	54.9 (14.7)	−1.2 (−9.8 to 7.4)	−0.1
Week 12	50.4 (11.7)	55.8 (19.0)	−4.4 (−15.2 to 6.4)	−0.3
Manic symptoms (YMRS)				
Week 0	14.6 (9.8)	13.9 (6.2)		
Week 2	9.4 (6.8)	11.2 (6.6)	−1.1 (−5.0 to 2.7)	−0.1
Week 4	8.1 (8.3)	7.8 (6.4)	0.0 (−4.4 to 4.4)	0.0
Week 12	5.4 (6.4)	7.8 (6.7)	−1.8 (−6.2 to 2.6)	−0.2
Global distress (CORE-10)				
Week 0	19.9 (8.4)	17.2 (9.9)		
Week 2	10.4 (5.6)	13.3 (7.4)	−3.7 (−6.9 to −0.5)	−0.4
Week 4	9.9 (6.4)	10.9 (6.6)	−1.9 (−5.0 to 1.2)	−0.2
Week 12	11.7 (8.0)	11.3 (6.2)	0.4 (−3.4 to 4.2)	0.0
Suicidal ideation (BSS)				
Week 0	4.6 (8.3)	6.7 (10.1)		
Week 2	0.8 (3.3)	3.6 (8.7)	−1.8 (−5.0 to 1.5)	−0.2
Week 4	1.1 (4.6)	3.0 (6.9)	−0.7 (−3.6 to 2.3)	−0.1
Week 12	1.3 (3.5)	2.0 (5.9)	0.4 (−3.3 to 4.2)	0.0
Duration of admission in days (baseline to 12 weeks)	32.5 (22.9)	37.9 (25.1)	−5.8 (−21.6 to 10.0)	−0.2
Duration of admission (baseline to discharge)	33.5 (25.6)	41.0 (33.7)	−8.5 (−28.0 to 11.1)	−0.3

Data are mean (s.d.).

PANSS, Positive and Negative Syndromes Scale; YMRS, Young Mania Rating Scale; CORE-10, Clinical Outcomes in Routine Evaluation, 10-item scale; BSS, Beck Suicide Scale.

All analyses controlled for baseline score for that variable and stratification factors (insomnia severity, diagnosis and wellbeing).

There was one SAE in the STAC group, a suicide attempt. This was deemed by the TSRG to be unrelated to participation in the study.

Eighty per cent (16 out of 20) of the participants who received therapy returned the Client Satisfaction Questionnaires (online Supplementary Table S4). Scores indicate a high level of satisfaction by the majority of participants. Nine of the 16 rated their overall satisfaction as ‘very satisfied’ and seven out of 16 rated their overall satisfaction as ‘mostly satisfied’. With regards to health-related quality of life (EQ-5D), there was a treatment benefit in the small effect size range for the STAC group when compared with SC. This was not maintained at follow-up (online Supplementary Table S5).

## Discussion

This is the first RCT of a psychological treatment for insomnia on an acute psychiatric inpatient ward setting. In this challenging environment for research, it was found that it is feasible to run an RCT of a psychological insomnia intervention. Despite experiencing acute psychiatric symptoms, participants were recruited with informed consent, completed assessments, were randomly allocated to two arms of the trial and the follow-up rate for assessments was high, even at 12 weeks when the majority of patients were discharged home. The rate of both uptake and completion of the therapy was 100% and patient satisfaction with the therapy was high.

Findings show that STAC may be highly beneficial for treating insomnia, with associated reductions in global distress at 2 weeks when compared with SC alone. Whilst the SC alone group showed improvement in insomnia across the 12-week assessment period, STAC led to a quicker and potentially fuller recovery from insomnia. The small effect on psychological wellbeing is promising. However, wide confidence intervals for the treatment effect include the possibility that there is no impact on wellbeing. Sub-group analysis revealed that patients with manic symptoms who had particularly elevated baseline wellbeing scores, which subsequently decreased, may have lessened the effect.

Providing psychological treatment for insomnia on an inpatient setting is a radical shift of focus. Sleep problems are pervasive on inpatient wards, despite the sedative effects of multiple medications (Waters *et al.* 2012), which is the common service response to patient reports of sleep difficulties (Rehman *et al.* 2016). The insomnia treatment produced a faster recovery than SC alone. ISI scores at week 2 for those who received the sleep treatment were equivalent to week 12 scores in the SC group. These results from an inpatient setting add to a growing body of research showing that it is feasible to treat sleep problems in patients with psychosis (Freeman *et al.* 2015) and bipolar affective disorder (Harvey *et al.* 2015) and, recently, patients at risk of psychosis (Bradley *et al.* in press).

Importantly, the therapy was also acceptable to the inpatient group: every patient who was offered STAC completed the full course of the therapy. The rationale of the study was that ‘*when people sleep better, they tend to feel better*’. We think this intuitive understanding of the importance of good sleep facilitated engagement, irrespective of insight into psychiatric symptoms. The intensive style of the therapy, providing an average of nine sessions over a 2-week period, was beneficial for supporting patients to try out new techniques. This intensity of the therapy may be particularly beneficial for sleep problems, whereby the sleep plan can be optimised each night in light of the previous night's sleep. The addition of sleep monitoring devices was both informative for the sleep plan and a way of acknowledging positive change to build patient motivation and self-efficacy.

It is possible that the therapy may have wider benefits beyond sleep, for example, the group that received STAC were discharged from hospital over a week (8.5 days) earlier than the SC group. However, the wide confidence intervals for the treatment effect include the possibility that there is no impact on length of admission. The trial was designed as a pilot study and hence the primary objective was to assess the feasibility of trial procedures, rather than assessing efficacy. A larger trial is now indicated, powered to detect plausible secondary effects of stabilising sleep.

A potential limitation of the trial is that it did not include objective measures of sleep (e.g. actigraphy or polysomnography) or relevant co-morbid sleep disorders (e.g. sleep apnoea or nightmare disorder). Our priority was to minimise assessment time and hence prioritise recruitment and retention of participants. Objective sleep assessments may also be less suitable for an inpatient group. Actigraphy infers sleep from a period of inactivity. Its accuracy for those with psychosis, who may be awake but inactive, has therefore been questioned (Freeman *et al.* 2015). Polysomnography involves sensors on the patient with connecting wires to transmit data. This equipment may pose a ligature risk and also further exacerbate sleep disruption. The male and predominantly white British sample may limit generalisability of results. A future study should recruit a more diverse group of inpatients. Given the potential causal association between insomnia and negative affect (Freeman *et al.* 2017), a future study may choose to include validated self-report measures of depression and anxiety. We compared the sleep treatment (in addition to SC) with SC alone, rather than an active control (e.g. befriending). This does not rule out the possibility that the efficacy effects are the result of unspecific aspects of the intensive therapy, rather than the STAC techniques specifically. Given this was a pilot RCT, our aim was to determine feasibility and collect initial efficacy data, rather than assessing what elements of the treatment produce change. The clear next step is a definitive trial powered to assess the efficacy of the intervention and the possible impact on both psychiatric symptoms and length of hospitalisation. If replicated, this inherently collaborative treatment approach for sleep could mark a radical shift of focus for psychiatric inpatient wards.

## Supplementary material

For supplementary material accompanying this paper visit https://doi.org/10.1017/S0033291717003191. click here to view supplementary material
