# Supplementary material for: Stabilising sleep for patients admitted at acute crisis to a psychiatric hospital (OWLS): an assessor-blind pilot randomised controlled trial
Source: Psychol Med. 2017 Nov 7;48(10):1694–704. doi: 10.1017/S0033291717003191 (PMC6088775; doi:10.1017/S0033291717003191)
Supplement: Supplementary file 1 [file S0033291717003191sup001.docx]

**SUPPLEMENTARY MATERIAL**

**Contents:**

1. **Table 1. PRN medication use over time.**
2. **Table 2. Sensitivity analysis of primary efficacy outcomes.**
3. **Table 3. Description of rationale for completing sub-group analysis on the primary efficacy outcome measures, splitting by baseline mania symptoms. Results of subgroup analysis and description of key findings.**
4. **Table 4. End of therapy client satisfaction questionnaire results.**
5. **Table 5. Scores for tertiary efficacy outcomes and description of key findings.**
6. **Sample size calculation for definitive trial of STAC versus standard care.**

**Supplementary material 1:**

Table 1. PRN MEDication use over time.

|  | | **Sleep treatment at acute crisis**  **(n=20)** | **Standard care (n=20)** |
| --- | --- | --- | --- |
| Number prescribed PRN anxiolytics | |  |  |
|  | Week 0 | 6 (30%) | 9 (45%) |
|  | Week 2 | 7 (35%) | 5 (25%) |
|  | Week 4 | 7 (35%) | 6 (30%) |
|  | Week 12 | 4 (20%) | 3 (15%) |
| Number prescribed a hypnotic medication | |  |  |
|  | Week 0 | 7 (35%) | 6 (30%) |
|  | Week 2 | 5 (25%) | 5 (25%) |
|  | Week 4 | 4 (20%) | 3 (15%) |
|  | Week 12 | 0 (0%) | 1 (5%) |
| Number prescribed PRN antipsychotics | |  |  |
|  | Week 0 | 4 (20%) | 2 (10%) |
|  | Week 2 | 4(20%) | 0 (0%) |
|  | Week 4 | 0 (0%) | 0 (0%) |
|  | Week 12 | 0 (0%) | 0 (0%) |

PRN=pro re nata (taken when necessary). Data are n (%).

**Supplementary material 2:**

Table 2. Sensitivity analysis of primary efficacy outcomes

|  | |  | **Sleep treatment at acute crisis**  **(n=20)** | **Standard care (n=20)** | **Adjusted mean difference between groups (95% CI)** | **Between group standardised effect size (d)** |
| --- | --- | --- | --- | --- | --- | --- |
| **Primary outcome measures**  Insomnia (ISI) | | |  |  |  |  |
|  | Week 2 | | 8.5 (5.4) | 12.5 (5.5) | -3.9 (-10.2; 2.3) | -0.7 |
| Wellbeing (WEMWBS) | | |  |  |  |  |
|  | Week 2 | | 47.4 (10.5) | 44.8 (13.4) | 3.0 (-5.7;11.8) | 0.2 |

Data are mean (SD). All analyses controlled for baseline score for that variable, stratification factors (insomnia severity, diagnosis and wellbeing) and baseline duration of admission.

**Supplementary material 3:**

Acute manic symptoms (including elevated mood and inflated self-esteem) were expected to impact on self-reported measures of symptoms, particularly wellbeing. Given that some, but not all, participants experienced symptoms of mania, sub-group analysis was planned splitting participants by the presence or absence of self-reported manic symptoms at baseline (score of ≥20 on baseline YMRS). At baseline, those with acute manic symptoms scored over 10 points higher for psychological wellbeing in both the treatment and control group. Hence it is highly plausible that decreasing or stable psychological wellbeing scores may reflect a positive change when a patient exhibits manic symptoms.

Table 3. Primary efficacy outcome measures split by baseline mania symptoms.

|  | | | |  | **Sleep treatment at acute crisis** | **Standard care** |
| --- | --- | --- | --- | --- | --- | --- |
| **Primary efficacy outcome measures**  Insomnia (ISI) | | | | | |  |
|  | Mania (n=7) | | | |  |  |
|  |  | Week 0 | | | 16.5(5.9) | 13.7 (5.7) |
|  |  | Week 2 | | | 9.3 (7.0) | 12.3 (5.8) |
|  |  | Week 4 | | | 5.5 (4.2) | 12.0 (4.0) |
|  |  | Week 12 | | | 1.7 (1.5) | 9.5 (4.9) |
|  | No mania (n=33) | | | |  |  |
|  |  | | Week 0 | | 17.3 (6.2) | 16.5 (4.8) |
|  |  | | Week 2 | | 8.3 (5.2) | 12.6 (5.6) |
|  |  | | Week 4 | | 7.1 (5.5) | 9.7 (5.8) |
|  |  | | Week 12 | | 6.8 (5.0) | 8.5 (4.5) |
| Wellbeing (WEMWBS) | | | | |  |  |
|  | Mania (n=7) | | | |  |  |
|  |  | | Week 0 | | 54.8 (12.4) | 52.7 (13.1) |
|  |  | | Week 2 | | 52.0 (14.5) | 57.7 (6.8) |
|  |  | | Week 4 | | 56.3 (5.9) | 49.7 (5.5) |
|  |  | | Week 12 | | 58.3 (10.2) | 53.0 (17.0) |
|  | No mania (n=33) | | | |  |  |
|  |  | | Week 0 | | 36.1 (14.0) | 40.5 (12.6) |
|  |  | | Week 2 | | 46.3 (9.6) | 42.5 (13.1) |
|  |  | | Week 4 | | 46.2 (12.1) | 44.9 (10.8) |
|  |  | | Week 12 | | 46.0 (11.9) | 43.4 (12.6) |

Data are mean (SD). Definition of mania = score of ≥20 on baseline YMRS.

For the group experiencing a manic episode at baseline, wellbeing scores had a different trajectory depending on allocation. The mean wellbeing scores for those who received standard care alone (n=3) increased from 52.7 (baseline, SD=13.1) to 57.7 (SD=6.8) at week 2. For those who received STAC (n=4) wellbeing scores remained relatively stable at 54.8 (baseline, SD=12.4) and 52.0 (week 2, SD=14.5). The opposite pattern was seen for those without manic symptoms. In the STAC group wellbeing scores increased from a baseline mean of 36.1 (SD=14.0) to 46.3 (SD=9.6) at week 2. However in the standard care alone group wellbeing scores remained more stable at 40.5 (baseline, SD=12.6) and 42.5 (week 2, SD=13.1). Acute manic symptoms may therefore be associated with elevated psychological wellbeing, which decreases in response to sleep treatment. For those without manic symptoms, wellbeing scores would be expected to increase in response to treatment. Acute manic symptoms did not affect the trajectory of change in insomnia symptoms, global distress, or suicidal ideation.

**Supplementary material 4:**

Table 4. Client satisfaction questionnaire (n=16)

| Questionnaire item | Scale rating | | | | |
| --- | --- | --- | --- | --- | --- |
| How would you rate the quality of the therapy that you have received? | Very poor  *n = 0* | Poor  *n = 0* | Fair  *n = 0* | Good  *n = 6* | Excellent  *n = 10* |
| Did you get the kind of therapy that you wanted? | No, definitely not  *n = 0* | No, not really  *n = 0* | Somewhat  *n = 1* | Yes, generally  *n = 5* | Yes, definitely  *n = 10* |
| If a friend were in need of similar help, would you recommend the programme? | No, definitely not  *n = 0* | No, probably not  *n = 0* | Unsure  *n = 0* | Yes, probably  *n = 3* | Yes, definitely  *n = 13* |
| How satisfied are you with the amount of therapy that you have received? | Quite dissatisfied  *n = 0* | Mildly dissatisfied  *n = 0* | Somewhat satisfied  *n = 1* | Mostly satisfied *n = 7* | Very satisfied  *n = 8* |
| Has the therapy helped you to deal more effectively with your problems? | No, it hasn’t helped at all  *n = 0* | No, it didn’t really help  *n = 0* | Unsure if it has helped  *n = 1* | Yes, it has helped  *n = 6* | Yes, it’s helped a great deal  *n = 9* |
| In an overall, general sense, how satisfied are you with the therapy you have received? | Quite dissatisfied  *n = 0* | Mildly dissatisfied  *n = 0* | Somewhat satisfied  *n = 0* | Mostly satisfied  *n = 7* | Very satisfied  *n = 9* |
| If you were to seek help again, would you come back to our programme? | No, definitely not  *n = 0* | No, I don’t think so  *n = 0* | Unsure  *n = 0* | Yes, I think so  *n = 4* | Yes, definitely  *n = 11* |

**Supplementary material 5:**

Table 5. Scores for Tertiary efficacy outcome measures

|  | |  | **Sleep treatment at acute crisis**  **(n=20)** | **Standard care (n=20)** | **Adjusted mean difference between groups (95% CI)** | **Standardised between group effect size (d)** |
| --- | --- | --- | --- | --- | --- | --- |
| Health related quality of life (EQ-5D) | | | |  |  |  |
|  | Week 0 | | 4.4 (2.9) | 3.8 (3.3) |  |  |
|  | Week 2 | | 2.3 (2.7) | 2.9 (3.4) | -0.8 (-2.5; 0.9) | -0.3 |
|  | Week 4 | | 2.3 (2.1) | 1.7 (2.1) | 0.3 (-0.9; 1.5) | 0.1 |
|  | Week 12 | | 2.6 (3.6) | 1.8 (2.1) | 0.7 (-1.4; 2.7) | 0.2 |

Data are mean (SD).

There was a small effect size improvement in health related quality of life (EQ-5D) in the STAC group at week 2. This was not sustained at week 4 and 12. At week 12 the standard care group had a small effect size benefit over the STAC group in terms of health related quality of life.

**Supplementary material 6:**

**Sample size calculation for a definitive trial of STAC plus standard care versus standard care alone.**

A sample size of 128 (64 per group) would allow for the detection of a treatment difference of 4.6 in the ISI score with 90% power and at a significance level of 5%, assuming a standard deviation of 7.2 and a dropout rate of 20%. The estimate of the standard deviation was determined as the maximum of the 80% confidence interval upper limits for the standard deviation of the ISI scores at the different assessment points.
